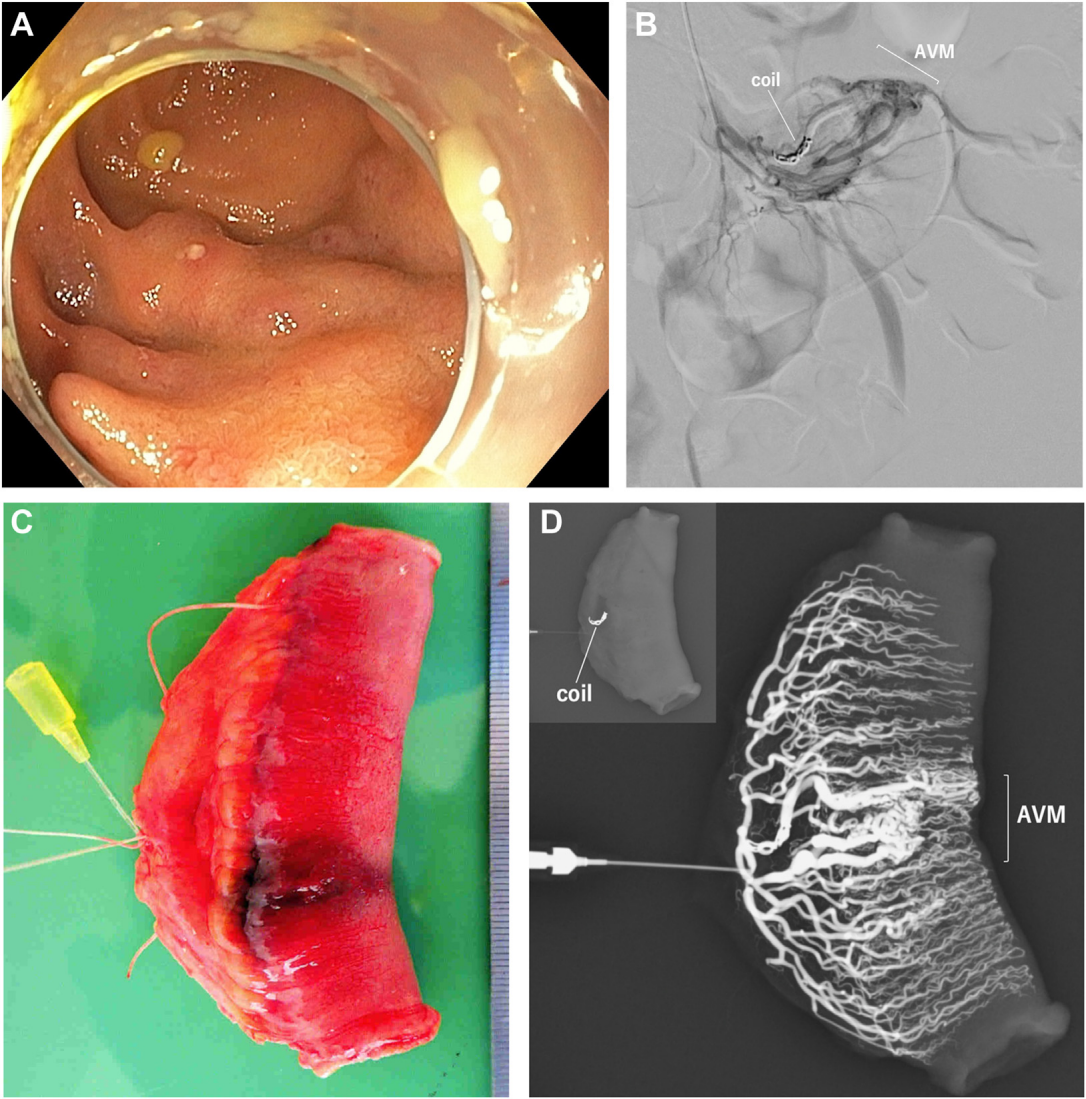# Challenges in Identifying and Resecting Small Bowel Arteriovenous Malformation

**DOI:** 10.1016/j.gastha.2024.03.004

**Published:** 2024-03-12

**Authors:** Shiho Nakamura, Naonori Inoue, Koji Uno

**Affiliations:** Department of Gastroenterology, Kyoto Second Red Cross Hospital, Kyoto, Japan

A 20-year-old woman presented to the outpatient clinic with a chief complaint of bloody stool. This time an enteroscopy revealed meandering vessels in the ileum that were suspected to be an arteriovenous malformation (Figure A). Angiography was performed to identify the lesion and evaluate the abnormal vessels. Coiling of the same vessel was performed for marking purposes (Figure B). A laparoscopic partial resection of the small intestine was performed. The intestinal tract with the lesion also showed vascular dilatation on the serosal surface. The implanted coil was palpable. The intestinal tract was resected to include the dilated vessel and the indwelling coil (Figure C). Contrast medium was injected into the resected intestinal vessels, and resection of the abnormal vessels was confirmed (Figure D). The patient’s postoperative course was good, and she was discharged on postoperative day 6.

Intraoperative localization of small bowel arteriovenous malformations can be difficult. The pathology may demonstrate the presence of abnormal vessels, but it is difficult to obtain a complete picture for the abnormal resected vessels. Thus, injection of contrast medium into the resected intestinal tract using fluoroscopy immediately after resection can confirm that the extent of resection was adequate.

**Figure:**